# The mental health burden of racial and ethnic minorities during the COVID-19 pandemic

**DOI:** 10.1371/journal.pone.0271661

**Published:** 2022-08-10

**Authors:** Long H. Nguyen, Adjoa Anyane-Yeboa, Kerstin Klaser, Jordi Merino, David A. Drew, Wenjie Ma, Raaj S. Mehta, Daniel Y. Kim, Erica T. Warner, Amit D. Joshi, Mark S. Graham, Carole H. Sudre, Ellen J. Thompson, Anna May, Christina Hu, Solveig Jørgensen, Somesh Selvachandran, Sarah E. Berry, Sean P. David, Maria Elena Martinez, Jane C. Figueiredo, Anne M. Murray, Alan R. Sanders, Karestan C. Koenen, Jonathan Wolf, Sebastien Ourselin, Tim D. Spector, Claire J. Steves, Andrew T. Chan

**Affiliations:** 1 Clinical and Translational Epidemiology Unit, Massachusetts General Hospital and Harvard Medical School, Boston, MA, United States of America; 2 Division of Gastroenterology, Massachusetts General Hospital and Harvard Medical School, Boston, MA, United States of America; 3 Department of Biostatistics, Harvard T.H. Chan School of Public Health, Boston, MA, United States of America; 4 School of Biomedical Engineering & Imaging Sciences, King’s College London, London, United Kingdom; 5 Diabetes Unit and Center for Genomic Medicine, Massachusetts General Hospital and Harvard Medical School, Boston, MA, United States of America; 6 Department of Medicine, Massachusetts General Hospital and Harvard Medical School, Boston, MA, United States of America; 7 Broad Institute of MIT and Harvard, Cambridge, MA, United States of America; 8 Department of Twin Research and Genetic Epidemiology, King’s College London, London, United Kingdom; 9 Harvard/MGH Center on Genomics, Vulnerable Populations, and Health Disparities, Massachusetts General Hospital and Harvard Medical School, Boston, MA, United States of America; 10 Zoe Ltd, London, United Kingdom; 11 Department of Nutritional Sciences, King’s College London, London, United Kingdom; 12 Department of Family Medicine, University of Chicago, Evanston, IL, United States of America; 13 Moores Cancer Center, University of California at San Diego, La Jolla, CA, United States of America; 14 Department of Family Medicine and Public Health, University of California at San Diego, La Jolla, CA, United States of America; 15 Department of Medicine, Samuel Oschin Comprehensive Cancer Institute, Cedars-Sinai Medical Center, Los Angeles, CA, United States of America; 16 Division of Geriatrics, Department of Medicine, Hennepin Healthcare, University of Minnesota, Minneapolis, MN, United States of America; 17 Berman Center for Outcomes and Clinical Research, Hennepin Healthcare Research Institute, Hennepin Healthcare, Minneapolis, MN, United States of America; 18 Department of Psychiatry and Behavioral Sciences, NorthShore University HealthSystem, Evanston, IL, United States of America; 19 Department of Psychiatry and Behavioral Neuroscience, University of Chicago, Chicago, IL, United States of America; 20 Department of Epidemiology, Harvard T.H. Chan School of Public Health, Boston, MA, United States of America; 21 Department of Immunology and Infectious Disease, Harvard T.H. Chan School of Public Health, Boston, MA, United States of America; 22 Massachusetts Consortium on Pathogen Readiness, Cambridge, MA, United States of America; Brown University, UNITED STATES

## Abstract

Racial/ethnic minorities have been disproportionately impacted by COVID-19. The effects of COVID-19 on the long-term mental health of minorities remains unclear. To evaluate differences in odds of screening positive for depression and anxiety among various racial and ethnic groups during the latter phase of the COVID-19 pandemic, we performed a cross-sectional analysis of 691,473 participants nested within the prospective smartphone-based COVID Symptom Study in the United States (U.S.) and United Kingdom (U.K). from February 23, 2021 to June 9, 2021. In the U.S. (*n*=57,187), compared to White participants, the multivariable odds ratios (ORs) for screening positive for depression were 1·16 (95% CI: 1·02 to 1·31) for Black, 1·23 (1·11 to 1·36) for Hispanic, and 1·15 (1·02 to 1·30) for Asian participants, and 1·34 (1·13 to 1·59) for participants reporting more than one race/other even after accounting for personal factors such as prior history of a mental health disorder, COVID-19 infection status, and surrounding lockdown stringency. Rates of screening positive for anxiety were comparable. In the U.K. (*n*=643,286), racial/ethnic minorities had similarly elevated rates of positive screening for depression and anxiety. These disparities were not fully explained by changes in leisure time activities. Racial/ethnic minorities bore a disproportionate mental health burden during the COVID-19 pandemic. These differences will need to be considered as health care systems transition from prioritizing infection control to mitigating long-term consequences.

## Introduction

The coronavirus disease-2019 (COVID-19) pandemic has led to over 178 million infections and 3.8 million deaths across the globe since December 2019 [1]. Widespread lockdowns forcing social isolation, coupled with widespread economic uncertainty [2,3] and the emotional toll of acute and chronic illness on those afflicted and their loved ones may have a negative long-term impact on mental health and well-being. Early work has demonstrated increased mental health symptoms linked to depression, and anxiety, as well as more frequent rates of substance abuse and suicide [2,4,5].

In the U.S., approximately 40% of the population is comprised of racial and ethnic minorities (i.e., non-White individuals), and in the U.K., this represents approximately 14% of the population [6,7]. Minority communities have disproportionately borne the burden of COVID-19, and have consistently had the highest rates of infection, severe disease, hospitalization and death from the virus [8,9]. These racial and ethnic disparities in COVID-19 in the U.S. and the U.K. reflect long-standing structural inequities and institutional racism in both countries, including discriminatory urban planning that has resulted in segregation into higher density housing and economic forces that have identified certain occupations as essential, and thus exempt from policies that might otherwise allow for greater social distancing [10–14].

Consequently, communities of color have been adversely impacted by the public health measures aimed at reducing the spread of COVID-19, including more frequent exemptions from mandatory lockdowns. Thus, we sought to assess the impact of the COVID-19 pandemic on odds of screening positive for depression and anxiety among a diverse cohort of individuals in the U.S. and the U.K.

## Materials and methods

### Study design and participants

We performed a cohort study in the U.S. and U.K. using the COVID Symptom Study (CSS) smartphone application. The application (“app”), developed by Zoe Global Ltd. in collaboration with researchers at the Massachusetts General Hospital, King’s College London, Lund University, and Uppsala University, has previously been described in detail [15]. In brief, the app was launched in the U.K. and U.S. in March 2020. Participants were initially recruited through social media outreach, invitations from the investigators of long-running cohort studies to their volunteers, and from the general public through the Apple App Store and Google Play Store.

The CSS app offers participants a guided interface to report baseline demographic information and relevant comorbidities, as well as information on potential COVID-19 symptoms and testing results. Participants are prompted to log daily, even when asymptomatic, for the longitudinal collection of incident symptoms and COVID-19 testing results. At enrollment, participants provided written consent to the use of information for research and agreed to applicable privacy policies and terms of use. This research study was approved by the Mass General Brigham Human Research Committee (Institutional Review Board Protocol 2020P000909) and King’s College London Ethics Committee (REMAS ID 18210).

### Choice of primary measure and ascertainment of mental health outcomes

Beginning February 23, 2021, we introduced a supplemental mental health questionnaire to U.K. and U.S. CSS app users, which we offered to all active users at the time of release. To maximize participation while minimizing user burden, our primary outcomes were based on the validated 4-item Patient Health Questionnaire for Depression and Anxiety (PHQ-4) [16], a brief screening tool for which two items from the longer 7-item General Anxiety Disorder (GAD-7) instrument and two from the 9-item Personal Health Questionnaire (PHQ-9) for depression were used to identify at-risk individuals.

The two questions for depression (PHQ-2) and two for anxiety (GAD-2) that were incorporated into the PHQ-4 have previously been demonstrated to account for 84% of the total variance, i.e. discriminating abilities, of the longer-form instruments from which they were derived and were strongly associated with functional impairment, disability days, healthcare usage, and functional status [16]. The questions (**S1 Table**) have four identical responses related to the frequency of depression/anxiety symptoms over the past 2 weeks: Not at all, several days, more than half the days, nearly every day. Responses are then scored on a Likert/ordinal scale (i.e., Not at all = 0 and nearly every day = 3) with a summed score greater than or equal to 3 by domain (e.g., depression or anxiety) representing a positive screen. In addition to PHQ-2 and GAD-2 scores, we queried whether participants had ever previously been diagnosed with a mental health condition and whether their pre- vs. peri-pandemic leisure time activities had changed (**S1 Table**).

### Ascertainment of racial/ethnic identity

Information collected using the CSS application has previously been provided [15]. Briefly, at the time of download/study enrollment, participants were asked with which race and/or ethnicity they self-identified based on standardized categories from the National Institutes of Health (NIH) in the U.S. [17] and the Office for National Statistics in the U.K. (**S2 Table**) [18]. In the U.S., Hispanic classification was defined as any race of Hispanic or Latino ancestry. Non-Hispanic categories were defined as each respective race not of Hispanic or Latino ancestry. Responses were then aggregated in a manner consistent with prior analyses. In both countries, those who identified as “Mixed Race” or selected more than one race were categorized as “More than one race”. We excluded individuals who selected “Prefer not to say” as their response or did not answer these questions (9% of respondents).

### Ascertainment of other covariates

We collected information on age (years), sex at birth (male, female, or other), weight (kg) and height (meters) were used to calculate body mass index (BMI, <18.5, 18.5-24.9, 25-29.9, and ≥30 kg/m^2^), prior history of diabetes, heart disease, lung disease, kidney disease, or active malignancy (each yes/no), smoking history (current/prior vs. never), and frontline HCW status (yes/no). We longitudinally ascertained whether they had ever tested positive for COVID-19 (yes/no), which was previously shown to have excellent agreement between self-report and confirmed test reports (**S1 Methods**).

To adjust for other regional factors, we linked volunteered participant location data (i.e., zip codes) to community-level socio-demographic indices related to education and income, as well the Oxford COVID-19 Government Response Tracker’s stringency index [19], a published resource to capture and score government policies related to public closure and containment, e.g., the strictness of lockdown policies designed to encourage social distancing.

### Statistical analysis

To investigate the determinants of mental health disparities during the COVID-19 pandemic, we performed multivariable logistic regression to estimate odds ratios (OR) for achieving a PHQ-2 or GAD-2 score ≥3 (yes/no) and their 95% confidence intervals (CIs) conditioned upon age, sex, and date of mental health questionnaire completion, adjusting for history of prior mental health diagnosis, diabetes, heart disease, lung disease, kidney disease, current/prior smoking status, BMI, prior history of COVID-19 infection, occupation as frontline HCW, geographic region (U.S.)/country (U.K.), and socio-demographic factors based on community-level measures of educational and financial deprivation, as well as lockdown stringency. We also performed stratified analyses among relevant patient subgroups and analyses by geographical region in which the referent group was White persons of the same subgroup (e.g., in stratified analyses of Black HCWs, odds of screening positive for depression or anxiety were compared to those of White HCWs). Two-sided p-values <0.05 were considered statistically significant. All statistical analyses were performed using R 4.0.3 (Vienna, Austria) and packages from the Bioconductor 3.12 release.

## Results

### Participant characteristics

Between February 23, 2021 and June 9, 2021, we enrolled 691,473 individuals (*n*= 57,187 U.S. and *n*=643,286 U.K participants). In the U.S., White participants tended to be older, reside in communities with higher income and educational attainment, and more frequently reported a prior mental health diagnosis than non-White individuals (**Table 1**). Similar trends in education and income were observed in the U.K.

**Table 1:** Baseline characteristics of study participants by race and ethnicity according to country of enrollment.

	United States (n=57187)					United Kingdom (n=634286)				
	White	Black	Hispanic	Asian	Other	White	Black	South Asian	Middle East/East Asian	Other
**n**	50410	1664	2239	2157	717	614902	3669	6231	2885	6599
**Age (years)**	61·7 (13·7)	60·5 (13·9)	52·4 (17·0)	58·3 (19·1)	59·0 (15·3)	56·6 (13·2)	50·5 (13·2)	50·8 (13·8)	50·7 (14·3)	50·2 (13·8)
<25	654 (1·3)	45 (2·7)	138 (6·2)	163 (7·6)	18 (2·5)	7451 (1·2)	117 (3·2)	166 (2·7)	72 (2·5)	198 (3·0)
25-34	2164 (4·3)	59 (3·5)	269 (12·0)	186 (8·6)	41 (5·7)	35342 (5·7)	399 (10·9)	643 (10·3)	358 (12·4)	761 (11·5)
35-44	4095 (8·1)	115 (6·9)	332 (14·8)	219 (10·2)	83 (11·6)	74032 (12·0)	600 (16·4)	1311 (21·0)	600 (20·8)	1358 (20·6)
45-54	5723 (11·4)	234 (14·1)	402 (18·0)	252 (11·7)	98 (13·7)	126942 (20·6)	975 (26·6)	1652 (26·5)	683 (23·7)	1591 (24·1)
55-64	11223 (22·3)	469 (28·2)	454 (20·3)	268 (12·4)	155 (21·6)	180790 (29·4)	1110 (30·3)	1340 (21·5)	601 (20·8)	1618 (24·5)
65-74	19583 (38·8)	535 (32·2)	448 (20·0)	518 (24·0)	230 (32·1)	152242 (24·8)	380 (10·4)	887 (14·2)	464 (16·1)	895 (13·6)
≥75	6968 (13·8)	207 (12·4)	196 (8·8)	551 (25·5)	92 (12·8)	38103 (6·2)	88 (2·4)	232 (3·7)	107 (3·7)	178 (2·7)
**Female sex**	37566 (74·5)	1394 (83·8)	1551 (69·3)	1382 (64·1)	514 (71·7)	410263 (66·7)	2499 (68·1)	3737 (60·0)	1833 (63·5)	4545 (68·9)
**BMI (kg/m** ^**2**^ **)**	27·1 (6·3)	30·0 (7·1)	28·0 (6·6)	25·2 (5·3)	28·4 (689)	26·8 (6·3)	28·5 (7·8)	25·7 (6·1)	25·4 (6·3)	26·6 (7·2)
<18.5	892 (1·8)	18 (1·1)	52 (2·3)	74 (3·4)	14 (2·0)	12203 (2·0)	80 (2·2)	259 (4·2)	104 (3·6)	181 (2·7)
18·5-24·9	20940 (41·5)	392 (23·6)	774 (34·6)	1147 (53·2)	224 (31·2)	263631 (42·9)	1252 (34·1)	3058 (49·1)	1545 (53·6)	3109 (47·1)
25-29·9	16272 (32·3)	541 (32·5)	728 (32·5)	638 (29·6)	260 (36·3)	206401 (33·6)	1208 (32·9)	1920 (30·8)	823 (28·5)	1971 (29·9)
≥30	12306 (24·4)	713 (42·8)	685 (30·6)	298 (13·8)	219 (30·5)	132667 (21·6)	1129 (30·8)	994 (16·0)	413 (14·3)	1338 (20·3)
**Comorbidities**										
Diabetes	2376 (4·7)	189 (11·4)	132 (5·9)	177 (8·2)	48 (6·7)	20470 (3·3)	182 (5·0)	449 (7·2)	100 (3·5)	235 (3·6)
Heart Disease	3560 (7·1)	110 (6·6)	122 (5·4)	166 (7·7)	58 (8·1)	22433 (3·6)	99 (2·7)	288 (4·6)	100 (3·5)	202 (3·1)
Lung Disease	1414 (2·8)	47 (2·8)	35 (1·6)	40 (1·9)	29 (4·1)	12627 (2·1)	64 (1·7)	111 (1·8)	54 (1·9)	111 (1·7)
Kidney Disease	901 (1·8)	41 (2·5)	39 (1·7)	48 (2·2)	20 (2·8)	5524 (0·9)	47 (1·3)	71 (1·1)	27 (0·9)	49 (0·7)
Cancer	1389 (2·8)	43 (2·6)	46 (2·1)	48 (2·2)	17 (2·4)	7218 (2·0)	45 (2·2)	55 (1·7)	20 (1·4)	52 (1·6)
**Education**	45·9 (18·4)	35·9 (17·6)	40·1 (19·0)	46·7 (17·6)	43·9 (19·0)	7·2 (2·5)	6·6 (2·6)	7·38 (2·45)	7·63 (2·32)	7·41 (2·40)
**Income**	812881 (314746)	67947 (286326)	761936 (312160)	89695 (31792)	777624 (290122)	7·0 (2·5)	5·64 (2·70)	6·5 (2·6)	6·6 (2·6)	6·5 (2·6)
**Lockdown stringency**	59·0 (7·5)	57·9 (6·5)	58·6 (7·0)	64·6 (9·1)	59·6 (8·0)	86·4 (3·0)	86·3 (3·2)	86·4 (3·1)	86·5 (3·0)	86·3 (3·2)
**Current/prior smoking**	14520 (28·8)	474 (28·5)	577 (25·8)	548 (25·4)	247 (34·4)	125141 (20·4)	773 (21·1)	841 (13·5)	493 (17·1)	1314 (19·9)
**Healthcare worker**	3957 (7·8)	137 (8·2)	195 (8·7)	152 (7·0)	56 (7·8)	24837 (4·0)	209 (5·7)	440 (7·1)	152 (5·3)	254 (3·8)
**Prior Covid-19**	2684 (5·3)	119 (7·2)	255 (11·4)	89 (4·1)	41 (5·7)	33727 (5·5)	343 (9·3)	537 (8·6)	210 (7·3)	489 (7·4)
**Prior mental health diagnosis**	14457 (28·7)	379 (22·8)	637 (28·5)	375 (17·4)	259 (36·2)	132514 (21·6)	875 (23·9)	930 (14·9)	432 (15·0)	1591 (24·1)

In the U.S., all racial categories were defined as each respective race not of Hispanic or Latino ancestry, and census-level data on education assessed the proportion of the general population above age 25 years with a Bachelor’s degree and income using median annual household income (U.S. dollars/year). In the U.K., census-level data on education used the education and income scores for the Index of Multiple Deprivation, respectively. Lockdown stringency was assessed using the Oxford COVID-19 Government Response Tracker’s index for overall government response at the time of mental health questionnaire completion. N (percentages) presented for categorical variables. Values are means (SD) for continuous variables. Values of polytomous variables may not sum to 100% due to rounding. Abbreviations: BMI (body mass index), m (meter), kg (kilogram).

### Racial and ethnic differences in depression and anxiety symptoms

In the U.S. and U.K., racial and ethnic minorities were more likely to screen positive for depression. In the U.S., compared to non-Hispanic White, the age-adjusted ORs for a PHQ-2 score ≥ 3 was 1·21 (95% CI: 1·07 to 1·37) for Black, 1·23 (1·12 to 1·36) for Hispanic participants, and 1·55 (95% CI: 1·32 to 1·83) for individuals reporting more than one/other race (**Table 2**).

**Table 2:** PHQ and GAD scores by race and ethnicity according to country of enrollment.

		**United States**				
		**White**	**Black**	**Hispanic**	**Asian**	**More than one/other**
**Number PHQ–2 ≥3 / total (%)**		6325/50410 (13%)	263/1664 (16%)	443/2239 (20%)	292/2157 (14%)	146/717 (20%)
**Age-adjusted OR (95% CI)** ^1^		1·0 [ref.]	1·21 (1·07 to 1·37)	1·23 (1·12 to 1·36)	0·92 (0·82 to 1·04)	1·55 (1·32 to 1·83)
**Multivariable-adjusted OR (95% CI)** ^2^		1·0 [ref.]	1·16 (1·02 to 1·31)	1·23 (1·11 to 1·36)	1·15 (1·02 to 1·30)	1·34 (1·13 to 1·59)
**Number GAD–2 ≥3 / total (%)**		7338/50410 (15%)	259/1664 (16%)	544/2239 (24%)	289/2157 (13%)	142/717 (20%)
**Age-adjusted OR (95% CI)** ^1^		1·0 [ref.]	1·01 (0·89 to 1·15)	1·17 (1·07 to 1·28)	0·73 (0·64 to 0·82)	1·23 (1·04 to 1·45)
**Multivariable-adjusted OR (95% CI)** ^2^		1·0 [ref.]	1·01 (0·89 to 1·14)	1·23 (1·12 to 1·35)	0·91 (0·81 to 1·03)	1·12 (0·94 to 1·32)
	**United Kingdom**					
	**White**		**Black**	**South Asian**	**Middle East/East Asian**	**More than one/other**
**Number PHQ–2 ≥3 / total (%)**	115277/614902 (19%)		890/3669 (24%)	1529/6231 (25%)	665/2885 (23%)	1477/6599 (22%)
**Age-adjusted OR (95% CI)** ^1^	1·0 [ref.]		1·18 (1·11 to 1·26)	1·20 (1·14 to 1·26)	1·11 (1·03 to 1·20)	1·07 (1·02 to 1·13)
**Multivariable-adjusted OR (95% CI)** ^2^	1·0 [ref.]		1·09 (1·01 to 1·17)	1·36 (1·29 to 1·44)	1·30 (1·20 to 1·41)	1·11 (1·05 to 1·17)
**Number GAD–2 ≥3 / total (%)**	107468/614902 (17%)		848/3669 (23%)	1402/6231 (23%)	585/2885 (20%)	1501/6599 (23%)
**Age-adjusted OR (95% CI)** ^1^	1·0 [ref.]		1·13 (1·06 to 1·21)	1·10 (1·04 to 1·15)	0·97 (0·89 to 1·05)	1·10 (1·04 to 1·15)
**Multivariable-adjusted OR (95% CI)** ^2^	1·0 [ref.]		1·07 (1·00 to 1·15)	1·26 (1·19 to 1·33)	1·11 (1·02 to 1·21)	1·11 (1·05 to 1·17)

^1^Conditioned upon age and date of mental health questionnaire completion.

^2^Additional conditioning upon sex and adjustment for personal history of mental health diagnosis, diabetes, heart disease, lung disease, kidney disease, current smoking status, body mass index, prior reported history of COVID-19 infection, and HCW status, as well as education, income, and lockdown stringency at the community level.

Abbreviations: CI (confidence interval), OR (odds ratio), PHQ-2 (Patient Health Questionnaire-2), GAD-2 (Generalized Anxiety Disorder 2-item).

Additional modeling adjusting for personal risk factors, including a prior history of mental health disease, community-level income, education, and local lockdown stringency somewhat attenuated our findings with a multivariable OR of 1·16 (95% CI: 1·02 to 1·31) for Black, 1·23 (1·11 to 1·36) for Hispanic participants, and 1·34 (95% CI: 1·13 to 1·59) for individuals reporting more than one/other race (**Table 2**). For Asian participants in the U.S., multivariable modeling strengthened, rather than weakened, the association between race and depressive symptoms, with an adjusted OR of 1·15 (1·02 to 1·30). We found comparably elevated odds of a positive depression screen among racial and ethnic minorities living in the U.K. compared to White persons.

With respect to anxiety symptoms, Hispanic individuals living in the U.S. had significantly greater odds of achieving a GAD-2 score ≥ 3 with a fully-adjusted OR of 1·23 (95% CI: 1·12 to 1·35). In the U.K., South Asian and Middle East/East Asian participants more frequently attained a positive screen for anxiety. In both countries, compared to depressive symptoms, race-based differences in reported anxiety symptoms were not as readily apparent with odds generally lower for anxiety compared to depression. In the U.S., we observed regional differences in the COVID-19-related mental health burden with the Northeast, South, and West at particularly high-risk of reporting symptoms of depression and anxiety; in the U.K, participants in Wales were disproportionately affected compared to other U.K. countries (**S3 Table**).

**Fig 1:**
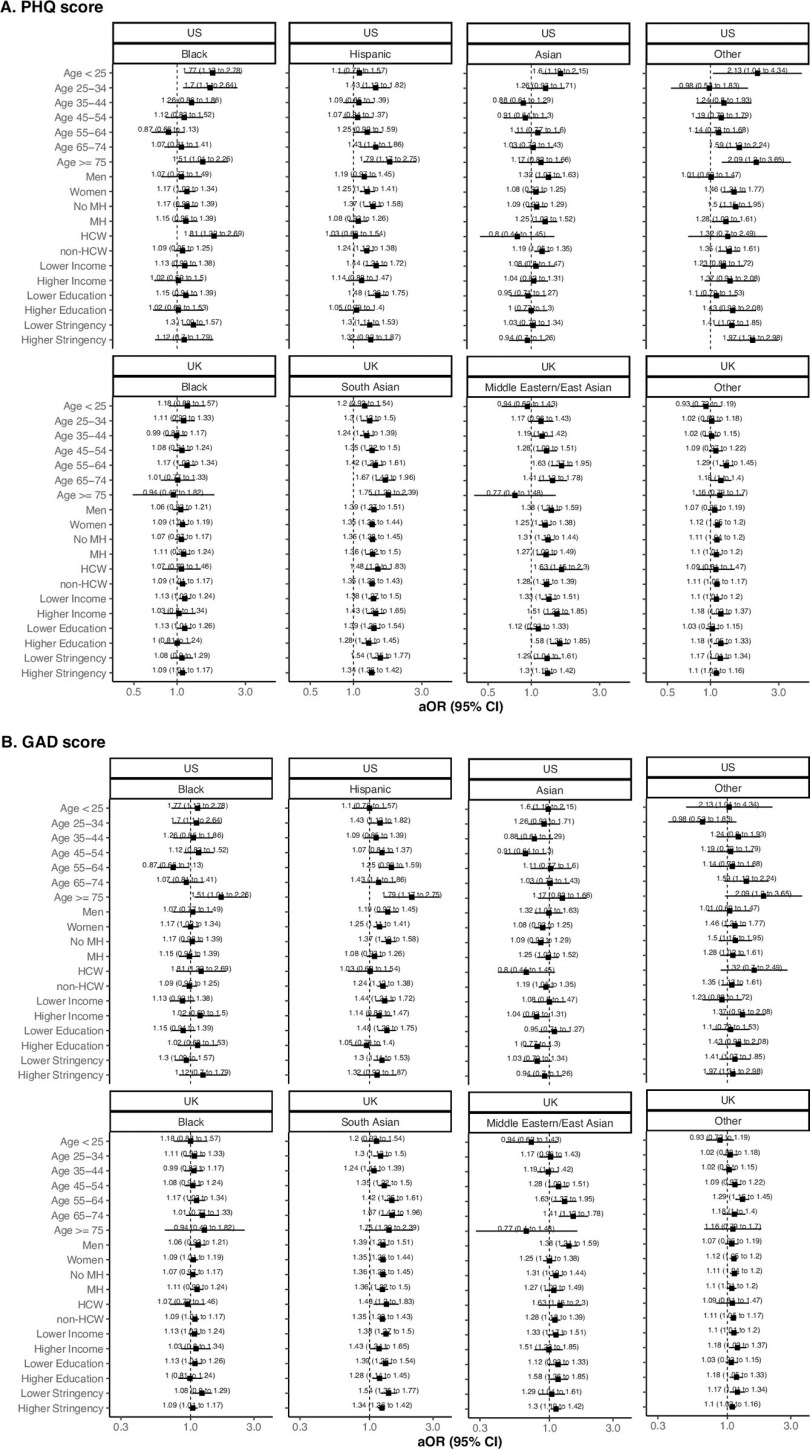
Odds of screening positive for depression or anxiety according to population subgroup. Stratified analyses for A. PHQ and B. GAD scores, respectively, each conditioned upon age, sex, and date of mental health questionnaire completion, and adjusted for personal history of mental health diagnosis, diabetes, heart disease, lung disease, kidney disease, current smoking status, body mass index, prior reported history of COVID-19 infection, HCW status, and community-level education, income, and lockdown stringency except in a given strata. Referent is White individuals of the same subgroup. Abbreviations: CI (confidence interval), OR (odds ratio), PHQ-2 (Patient Health Questionnaire-2), GAD-2 (Generalized Anxiety Disorder 2-item).

**Fig 2:**
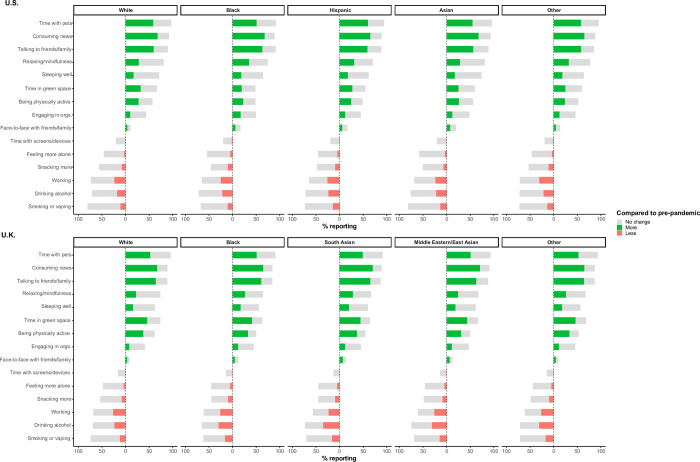
Changes in peri-pandemic behavior by race and ethnicity. Proportions have been rescaled among individuals to whom a given question was deemed applicable (i.e., time spent with pets reported among pet owners). Stratified analyses conditioned upon age, sex, and date of mental health questionnaire completion, and adjusted for personal history of mental health diagnosis, diabetes, heart disease, lung disease, kidney disease, current smoking status, body mass index, prior reported history of COVID-19 infection, HCW status, and community-level education, income, and lockdown stringency except in a given strata. Referent is White individuals of the same subgroup.

To evaluate whether the observed links between race and mental health outcomes during the COVID-19 pandemic differed based on population subgroups, we performed stratified analyses by sex, prior mental health history, frontline healthcare worker status, income, education, and lockdown stringency. In the U.S., we observed greater odds of screening positive for depression among Black HCWs compared to White HCWs (**Fig 1**). In general, odds of depression and anxiety tended to be higher among U.S. racial and ethnic minority individuals in the highest age group (≥75 years) compared to White persons of the same age group (**Fig 1**). In the U.S. and U.K., we found no consistent relationship between race, ethnicity, and depression/anxiety according to community-level lockdown stringency (**Fig 1**). No significant differences were observed among other subgroups assessed in both countries, and findings related to anxiety revealed similar within-group concordance. Similarly, racial and ethnic disparities in depression and anxiety were not reflected by differences in pre- vs. peri-pandemic leisure-time activities, including time spent with pets, working, smoking/vaping or drinking, nor time spent alone, which were grossly comparable across race/ethnicity groups in the U.S. and U.K. (**Fig 2**).

## Discussion

This analysis among a large population of individuals in the U.S. and U.K. showed racial and ethnic minorities were at greater odds of depression and anxiety in the midst of the COVID-19 pandemic. Compared to White HCWs, Black HCWs had higher odds of depression and anxiety symptoms. This trend was also observed among older racial and ethnic minority individuals compared to older White persons. There is a growing body of literature linking worsened mental health outcomes to the drastic disruptions in everyday life that have characterized the COVID-19 pandemic [20]. Specifically, a 3-fold increase in reported symptoms of depression was noted in the peri-pandemic compared to the pre-pandemic period^2^, with young adults, lower income individuals, and those with greater exposure to stressors and lower health literacy at particularly heightened risk [20,21]. Similarly, amongst college students, approximately 90% reported increased depressive symptoms and 60% noted increased anxiety symptoms during the pandemic [22].

Racial and ethnic mental health disparities, or differences in the prevalence rates, diagnoses, access to care and sources of care [23], have been noted in prior studies. Increased risk of formally-diagnosed anxiety in Hispanic individuals compared to Black respondents has been demonstrated in other investigations [24]. Further, higher risk of anxiety has been noted in Hispanic individuals with good or excellent English proficiency compared to those with lower proficiency [25]. These prior findings may help explain our observation of higher odds of anxiety symptoms in Hispanic individuals using our smartphone-based survey with no corresponding increase among other racial and ethnic minorities.

Racial and ethnic minorities face significant challenges related to the diagnosis and treatment of mental health disorders in the U.S. and the U.K. including poor access to mental healthcare services due to structural inequities and systematic disinvestment within minority communities [10,11,26], disparate treatment by providers who may minimize their symptoms due to implicit bias and racism, and even systematic differences in prescribed treatment choices.[27–29] Further, perceived discrimination is also associated with negative mental health outcomes for minorities in both the U.S. and U.K. [28], while the considerable stigma surrounding mental illness has long-been been established among Black and Hispanic populations [30–32].

Our study is strengthened by the population-scale enrollment of a diverse group of participants from two similarly afflicted nations using a common data collection instrument. Our binational study design offered a unique opportunity to consider the degree to which broad differences in nationalized care access could result in differences in the observed mental health burden of racial and ethnic minorities. Our digital platform to rapidly collect a validated instrument to assess risk of depression and anxiety can help provide real-time actionable insights, while our collection of extensive demographic and comorbidity information generally not available in registry-level data or large-scale surveillance efforts, with external linkage to community-level information on education, income, and lockdown stringency helped deepen our understanding of the observed mental health disparities.

We recognize several important limitations. As with all studies relying primarily on volunteered information, measurement and reporting bias are possible. However, our internal validation study (**S1 Methods**) demonstrates that self-reported information from the general population could be accurately and faithfully reported, particularly as it pertains to our primary outcome (perceived mental health symptoms). While we enrolled a comparatively lower proportion of racial and ethnic minorities compared to national censuses in both countries, there were relatively high absolute numbers for most groups allowing for statistically robust between-group comparisons [6,7]. To limit participant burden, greater detail on racial/ethnic self-identity was not obtained, prohibiting disaggregated analyses by subgroup, and our current categorizations may oversimplify or incompletely characterize the different experiences of minority participants in the U.S. and U.K. Despite employing similar strategies in the U.K. and the U.S. to recruit members of the general public and participants of long-running cohort studies, the enrolled population in the U.K. was much larger than the U.S. However, our sample size remained substantial in both countries, allowing for internally consistent country-specific estimates.

While greater than 80% of U.S. adults use smartphones [33], we acknowledge the COVID Symptom Study could have comparatively lower penetrance among certain socioeconomic/age groups. However, under-recruitment of more deprived or less technologically-literate participants would have attenuated observed differences, and we demonstrate that racial/ethnic disparities in mental health outcomes persisted despite uniform access to technology. Finally, our cohort of study volunteers willing to share information about mental health symptoms may not represent a random sampling of the U.S. and U.K. population, though our survey was available to all active participants, and the observed differences in depressive and anxiety symptoms among racial and ethnic minorities remain internally valid.

## Conclusion

In closing, we found a greater risk for symptoms of depression and anxiety amongst racial and ethnic minorities in the U.S. and the U.K. during the COVID-19 pandemic. Our findings highlight the urgency for open discussions surrounding mental health and wellness among minority communities disproportionately impacted by the COVID-19 pandemic who stand to suffer well beyond its presumed conclusion. Discussions around these topics may alleviate the stigma surrounding mental illness in such populations and ensure that those who need treatment are identified in a timely fashion.

## Supporting information

S1 Methods (DOCX)

S1 Table Mental health questions in the COVID Symptom Study. (DOCX)

S2 Table Race/Ethnicity categories by country of enrollment. (DOCX)

S3 Table PHQ and GAD scores by region. (DOCX)

## References

[1] COVID-19 map - johns Hopkins Coronavirus resource Center. [cited 21 Jun 2021]. Available: https://coronavirus.jhu.edu/map.html.

[2] Achdut N, Refaeli T. Unemployment and Psychological Distress among Young People during the COVID-19 Pandemic: Psychological Resources and Risk Factors. Int J Environ Res Public Health. 2020;17. doi: 10.3390/ijerph17197163 33007892 PMC7579061

[3] Berkowitz SA, Basu S. Unmet Social Needs And Worse Mental Health After Expiration Of COVID-19 Federal Pandemic Unemployment Compensation. Health Aff. 2021;40: 426–434. doi: 10.1377/hlthaff.2020.01990 33600235 PMC8053426

[4] Xiong J, Lipsitz O, Nasri F, Lui LMW, Gill H, Phan L, et al. Impact of COVID-19 pandemic on mental health in the general population: A systematic review. J Affect Disord. 2020;277: 55–64. doi: 10.1016/j.jad.2020.08.001 32799105 PMC7413844

[5] Ornell F, Moura HF, Scherer JN, Pechansky F, Kessler FHP, von Diemen L. The COVID-19 pandemic and its impact on substance use: Implications for prevention and treatment. Psychiatry Res. 2020;289: 113096. 10.1016/j.psychres.2020.113096 PMC7219362 32405115

[6] US Census Bureau. Population and Housing Unit Estimates. 2022 [cited 8 May 2022]. Available: https://www.census.gov/programs-surveys/popest.html.

[7] 2011 census. [cited 8 May 2022]. Available: https://www.ons.gov.uk/census/2011census.

[8] Gross CP, Essien UR, Pasha S, Gross JR, Wang S-Y, Nunez-Smith M. Racial and Ethnic Disparities in Population-Level Covid-19 Mortality. J Gen Intern Med. 2020;35: 3097–3099. doi: 10.1007/s11606-020-06081-w 32754782 PMC7402388

[9] Selden TM, Berdahl TA. COVID-19 And Racial/Ethnic Disparities In Health Risk, Employment, And Household Composition. Health Aff. 2020;39: 1624–1632. doi: 10.1377/hlthaff.2020.00897 32663045

[10] Bailey ZD, Feldman JM, Bassett MT. How Structural Racism Works - Racist Policies as a Root Cause of U.S. Racial Health Inequities. N Engl J Med. 2021;384: 768–773. doi: 10.1056/NEJMms2025396 33326717 PMC11393777

[11] Gee GC, Ford CL. STRUCTURAL RACISM AND HEALTH INEQUITIES: Old Issues, New Directions. Du Bois Rev. 2011;8: 115–132. doi: 10.1017/S1742058X11000130 25632292 PMC4306458

[12] Williams DR, Rucker TD. Understanding and addressing racial disparities in health care. Health Care Financ Rev. 2000;21: 75–90. 11481746 PMC4194634

[13] Hackett RA, Ronaldson A, Bhui K, Steptoe A, Jackson SE. Racial discrimination and health: a prospective study of ethnic minorities in the United Kingdom. BMC Public Health. 2020;20: 1652. doi: 10.1186/s12889-020-09792-1 33203386 PMC7672934

[14] Nguyen LH, Drew DA, Graham MS, Joshi AD, Guo C-G, Ma W, et al. Risk of COVID-19 among front-line health-care workers and the general community: a prospective cohort study. Lancet Public Health. 2020;5: e475–e483. doi: 10.1016/S2468-2667(20)30164-X 32745512 PMC7491202

[15] Drew DA, Nguyen LH, Steves CJ, Menni C, Freydin M, Varsavsky T, et al. Rapid implementation of mobile technology for real-time epidemiology of COVID-19. Science. 2020;368: 1362–1367. doi: 10.1126/science.abc0473 32371477 PMC7200009

[16] Kroenke K, Spitzer RL, Williams JBW, Löwe B. An ultra-brief screening scale for anxiety and depression: the PHQ-4. Psychosomatics. 2009;50: 613–621. doi: 10.1176/appi.psy.50.6.613 19996233

[17] NOT-OD-15-089: Racial and Ethnic Categories and Definitions for NIH Diversity Programs and for Other Reporting Purposes. [cited 3 May 2021]. Available: https://grants.nih.gov/grants/guide/notice-files/NOT-OD-15-089.html.

[18] Ethnic group, national identity and religion. [cited 3 May 2021]. Available: https://www.ons.gov.uk/methodology/classificationsandstandards/measuringequality/ethnicgroupnationalidentityandreligion.

[19] Hale T, Angrist N, Goldszmidt R, Kira B, Petherick A, Phillips T, et al. A global panel database of pandemic policies (Oxford COVID-19 Government Response Tracker). Nat Hum Behav. 2021;5: 529–538. doi: 10.1038/s41562-021-01079-8 33686204

[20] Reading Turchioe M, Grossman LV, Myers AC, Pathak J, Creber RM. Correlates of Mental Health Symptoms Among US Adults During COVID-19, March-April 2020. Public Health Rep. 2021;136: 97–106. doi: 10.1177/0033354920970179 33211985 PMC7856377

[21] Ettman CK, Abdalla SM, Cohen GH, Sampson L, Vivier PM, Galea S. Prevalence of Depression Symptoms in US Adults Before and During the COVID-19 Pandemic. JAMA Netw Open. 2020;3: e2019686. doi: 10.1001/jamanetworkopen.2020.19686 32876685 PMC7489837

[22] López-Castro T, Brandt L, Anthonipillai NJ, Espinosa A, Melara R. Experiences, impacts and mental health functioning during a COVID-19 outbreak and lockdown: Data from a diverse New York City sample of college students. PLoS One. 2021;16: e0249768. doi: 10.1371/journal.pone.0249768 33826654 PMC8026074

[23] Primm AB, Vasquez MJT, Mays RA, Sammons-Posey D, McKnight-Eily LR, Presley-Cantrell LR, et al. The role of public health in addressing racial and ethnic disparities in mental health and mental illness. Prev Chronic Dis. 2010;7: A20. 20040235 PMC2811515

[24] Vanderminden J, Esala JJ. Beyond symptoms: Race and gender predict anxiety disorder diagnosis. Soc Ment Health. 2019;9: 111–125.

[25] Alegría M, Mulvaney-Day N, Torres M, Polo A, Cao Z, Canino G. Prevalence of psychiatric disorders across Latino subgroups in the United States. Am J Public Health. 2007;97: 68–75. doi: 10.2105/AJPH.2006.087205 17138910 PMC1716243

[26] Bailey ZD, Krieger N, Agénor M, Graves J, Linos N, Bassett MT. Structural racism and health inequities in the USA: evidence and interventions. Lancet. 2017;389: 1453–1463. doi: 10.1016/S0140-6736(17)30569-X 28402827

[27] Memon A, Taylor K, Mohebati LM, Sundin J, Cooper M, Scanlon T, et al. Perceived barriers to accessing mental health services among black and minority ethnic (BME) communities: a qualitative study in Southeast England. BMJ Open. 2016;6: e012337. doi: 10.1136/bmjopen-2016-012337 27852712 PMC5128839

[28] Williams DR. Stress and the Mental Health of Populations of Color: Advancing Our Understanding of Race-related Stressors. J Health Soc Behav. 2018;59: 466–485. doi: 10.1177/0022146518814251 30484715 PMC6532404

[29] Chow JC-C, Jaffee K, Snowden L. Racial/ethnic disparities in the use of mental health services in poverty areas. Am J Public Health. 2003;93: 792–797. doi: 10.2105/ajph.93.5.792 12721146 PMC1447841

[30] Hines-Martin V, Malone M, Kim S, Brown-Piper A. Barriers to mental health care access in an African American population. Issues Ment Health Nurs. 2003;24: 237–256. doi: 10.1080/01612840305281 12623684

[31] DeFreitas SC, Crone T, DeLeon M, Ajayi A. Perceived and Personal Mental Health Stigma in Latino and African American College Students. Front Public Health. 2018;6: 49. doi: 10.3389/fpubh.2018.00049 29536000 PMC5834514

[32] Jimenez DE, Bartels SJ, Cardenas V, Alegría M. Stigmatizing attitudes toward mental illness among racial/ethnic older adults in primary care. Int J Geriatr Psychiatry. 2013;28: 1061–1068. doi: 10.1002/gps.3928 23361866 PMC3672370

[33] Demographics of mobile device ownership and adoption in the United States. 7 Apr 2021 [cited 11 May 2021]. Available: https://www.pewresearch.org/internet/fact-sheet/mobile/.

