# Supplementary material for: The mental health burden of racial and ethnic minorities during the COVID-19 pandemic
Source: PLoS One. 2022 Aug 10;17(8):e0271661. doi: 10.1371/journal.pone.0271661 (PMC9365178; doi:10.1371/journal.pone.0271661)
Supplement: S1 Methods — (DOCX) [file pone.0271661.s001.docx]

## Supplementary Materials and Methods

**Prior validation work**

We performed an internal validation study to ascertain the validity of self-reported COVID Symptom Study information among a random subset of 235 users who reported a COVID-19 test. We requested photographs of their COVID-19 test report, and study staff blinded to participant-provided information found excellent agreement between self-report and test reports with 88% sensitivity and 94% specificity (unpublished).

**Ascertainment of racial and ethnic identity**

Individuals who identified their race or ethnicity as “Other” were then presented a window for free-text entry. Those who identified as “Mixed Race” or selected more than one race were categorized as “More than one race”. In the U.S., “Native Hawaiian and Pacific Islanders” were classified as Asian. Due to limited sample sizes, “American Indian or Alaskan Natives” were categorized as “Other”, and “Other” and “More than one race” were combined. In the U.K., individuals were asked whether they identified as “Chinese” or “Asian/British Asian”, which offered the following examples, but did not specifically ask about other racial identities from the Asian continent: “Indian, Pakistani, Bangladeshi, Other”.

**Community-level sociodemographic factors**

Participants who elected to share information on their zip code (U.S./U.K.) of residence were assigned community-level socioeconomic measures. Socioeconomic measures were generated using established metrics derived from aggregated census data: proportion of individuals aged ≥25 years old with a Bachelor’s degree and median annual income (U.S.)[^31^](https://paperpile.com/c/gJR6OR/ZRID) and the education and income measures from the U.K.’s Indices of Multiple Deprivation (deciles, IMD),[^32^](https://paperpile.com/c/gJR6OR/9kxv) respectively. U.S. zip codes were further categorized into broader regions using U.S. Census Bureau criteria[^33^](https://paperpile.com/c/gJR6OR/LtjA) (American Northeast, Midwest, South, and West) and U.K. zip codes were linked to one of four countries (England, Scotland, Northern Ireland, and Wales).[^34^](https://paperpile.com/c/gJR6OR/jXtF)

**Supplementary statistical analysis, handling of missing data, and power**

For missing data, imputation replaced no more than 5% of missing values for a given metadatum with numeric values replaced with the median and categorical variables imputed using the mode. Complete case analyses without imputation were performed to ensure robustness of findings and to ensure correction of missingness did not materially alter risk estimates (data not shown). LHN, AAY, and ATC had access to raw data. LHN performed data analysis, and the corresponding author had full access to data and the final responsibility to submit for publication.

**Data Sharing**

Data collected using the COVID Symptom Study smartphone application are being shared with other health researchers through the U.K. National Health Service-funded Health Data Research UK (HDRUK) and Secure Anonymised Information Linkage consortium, housed in the U.K. Secure Research Platform (Swansea, U.K.). Anonymized data are available to be shared with researchers according to their protocols in the public interest (<https://web.www.healthdatagateway.org/dataset/fddcb382-3051-4394-8436-b92295f14259>). U.S. investigators are encouraged to coordinate data requests through the Coronavirus Pandemic Epidemiology (COPE) Consortium (<https://www.monganinstitute.org/cope-consortium>).

## 
