# Supplementary material for: The mental health burden of racial and ethnic minorities during the COVID-19 pandemic
Source: PLoS One. 2022 Aug 10;17(8):e0271661. doi: 10.1371/journal.pone.0271661 (PMC9365178; doi:10.1371/journal.pone.0271661)
Supplement: S1 Table — (DOCX) [file pone.0271661.s002.docx]

## Supplementary Table 1. Mental health questions in the COVID Symptom Study

| **About our history of mental health** |
| --- |
| Have you ever been diagnosed with a mental health condition? |
| Do you have learning difficulty (e.g., dyslexia)? |
| **Over the last 2 weeks, how often have you been bothered by the following for:** |
| PHQ-2 Questions |
| Little interest or pleasure in doing things |
| Feeling down, depressed, or hopeless |
| GAD-2 Questions |
| Feeling nervous, anxious, or on edge |
| Not able to stop or control worrying |
| *Responses for PHQ-2 and GAD-2 questions range scored on Likert/ordinal scale, with 0 = Not at all, 1 = several days, 2= more than half the days, 3 = nearly every day |
| **During this pandemic, have you changed the way you have spent time doing the following:** |
| Sleeping well |
| Being physically active/doing exercise |
| Spending time in green spaces such as parks, gardens, country side |
| Spending time with pets |
| Smoking or vaping |
| Drinking alcohol |
| Interacting face-to-face with family/friends |
| Talking to family/friends via phone/technology |
| Feeling more alone |
| Working |
| Relaxation/mindfulness/meditation |
| Reading/watching/listening to the news |
| Using devices with a screen |
| Eating savory snacks/confectionery |
| Engaging in organizations, clubs, or societies (e.g. political, religious, charitable, social, sport, or other groups, organizations, clubs, or societies) |
| *Responses for leisure-time activity were “more”, “less”, “no change”, or “not applicable” |
