# Supplementary material for: The mental health burden of racial and ethnic minorities during the COVID-19 pandemic
Source: PLoS One. 2022 Aug 10;17(8):e0271661. doi: 10.1371/journal.pone.0271661 (PMC9365178; doi:10.1371/journal.pone.0271661)
Supplement: S2 Table — (DOCX) [file pone.0271661.s003.docx]

## Supplementary Table 2. Race/ethnicity categories by country of enrollment

| **United States** | |
| --- | --- |
| **Responses** | **Categories** |
| Non-Hispanic White | White, non-Hispanic |
| Black or African-American | Black |
| Hispanic/LatinX | Hispanic |
| Asian | Asian |
| Native Hawaiian or other Pacific Islander | Asian |
| American Indian or Alaska Native | More than one/other |
| Other non-Hispanic racial group, other racial or ethnic group, multiple race/ethnicity groups identified | More than one/other |
| **United Kingdom** | |
| **Responses** | **Categories** |
| White- British, Irish, or other | White |
| Black/Black British - Caribbean, African, or other | Black |
| Asian/Asian British - Indian, Pakistani, Bangladeshi, or other | Middle East/East Asian |
| Chinese/Chinese British | Chinese |
| Middle Eastern/Middle Eastern British - Arab, Turkish, other | Middle East/East Asian |
| East/Southeast Asian, other, or mixed/multiple race - White and Black/Black British, other | More than one/other |

## 
