# Supplementary material for: The mental health burden of racial and ethnic minorities during the COVID-19 pandemic
Source: PLoS One. 2022 Aug 10;17(8):e0271661. doi: 10.1371/journal.pone.0271661 (PMC9365178; doi:10.1371/journal.pone.0271661)
Supplement: S3 Table — (DOCX) [file pone.0271661.s004.docx]

## Supplementary Table 3. PHQ and GAD scores by region

|  | **United States** | | | |
| --- | --- | --- | --- | --- |
|  | **Midwest** | **Northeast** | **South** | **West** |
| **Number PHQ–2 ≥3 / total** | 1213/10258 | 1683/12982 | 1948/13698 | 2381/18813 |
| **Age-adjusted OR (95% CI)^1^** | 1·0 (ref.) | 1·13 (1·05 to 1·21) | 1·23 (1·14 to 1·32) | 1·18 (1·11 to 1·27) |
| **Multivariable-adjusted OR (95% CI)^2^** | 1·0 (ref.) | 1·25 (1·15 to 1·35) | 1·23 (1·15 to 1·33) | 1·32 (1·22 to 1·42) |
|  | | | | |
| **Number GAD–2 ≥3 / total** | 1473/10258 | 1964/12982 | 2279/13698 | 2609/18813 |
| **Age-adjusted OR (95% CI)^1^** | 1·0 (ref.) | 1·09 (1·02 to 1·16) | 1·18 (1·11 to 1·27) | 1·12 (1·05 to 1·19) |
| **Multivariable-adjusted OR (95% CI)^2^** | 1·0 (ref.) | 1·18 (1·10 to 1·27) | 1·20 (1·12 to 1·28) | 1·21 (1·13 to 1·30) |
| ^1^Conditioned upon age and date of mental health questionnaire completion  ^2^Additional conditioning upon sex and adjustment for personal history of mental health diagnosis, diabetes, heart disease, lung disease, kidney disease, current smoking status, body mass index, prior reported history of COVID-19 infection, and HCW status, as well as education, income, and lockdown stringency at the community level  Regions defined by the U.S. Census Bureau guidelines. The Northeast included the states of Connecticut, Maine, Massachusetts, New Hampshire, New Jersey, New York, Pennsylvania, Rhode Island, and Vermont. The Midwest included the states of Illinois, Indiana, Iowa, Kansas, Michigan, Minnesota, Missouri, Nebraska, North Dakota, Ohio, South Dakota, and Wisconsin. The South included Alabama, Arkansas, Delaware, Florida, Georgia, Kentucky, Louisiana, Maryland, Mississippi, North Carolina, Oklahoma, South Carolina, Tennessee, Texas, Virginia, and West Virginia. The West included Alaska, Arizona, California, Colorado, Hawaii, Idaho, Montana, New Mexico, Nevada, Oregon, Utah, Washington, and Wyoming.  Abbreviations: CI (confidence interval), OR (odds ratio), PHQ-2 (Patient Health Questionnaire-2), GAD-2 (Generalized Anxiety Disorder 2-item) | | | | |

##

|  | **United Kingdom** | | | |
| --- | --- | --- | --- | --- |
|  | **England** | **Northern Ireland** | **Scotland** | **Wales** |
| **Number PHQ–2 ≥3 / total** | 100884/538924 | 656/3394 | 6535/33046 | 6206/27968 |
| **Age-adjusted OR (95% CI)^1^** | 1·0 (ref.) | 1·03 (0·95 to 1·11) | 1·07 (1·04 to 1·10) | 1·25 (1·22 to 1·29) |
| **Multivariable-adjusted OR (95% CI)^2^** | 1·0 (ref.) | 1·05 (0·96 to 1·15) | 1·09 (1·05 to 1·13) | 1·22 (1·17 to 1·27) |
|  | | | | |
| **Number GAD–2 ≥3 / total** | 94770/538924 | 629/3394 | 5824/33046 | 5281/27968 |
| **Age-adjusted OR (95% CI)^1^** | 1·0 (ref.) | 1·05 (0·97 to 1·14) | 1·01 (0·98 to 1·04) | 1·15 (1·11 to 1·18) |
| **Multivariable-adjusted OR (95% CI)^2^** | 1·0 (ref.) | 1·10 (1·01 to 1·20) | 1·02 (0·99 to 1·06) | 1·13 (1·08 to 1·18) |
| ^1^Conditioned upon age and date of mental health questionnaire completion  ^2^Additional conditioning upon sex and adjustment for personal history of mental health diagnosis, diabetes, heart disease, lung disease, kidney disease, current smoking status, body mass index, prior reported history of COVID-19 infection, and HCW status, as well as education, income, and lockdown stringency at the community level  Abbreviations: CI (confidence interval), OR (odds ratio), PHQ-2 (Patient Health Questionnaire-2), GAD-2 (Generalized Anxiety Disorder 2-item) | | | | |
